# Diagnosing Appendicitis in Pregnancy Via Ultrasonography

**DOI:** 10.7759/cureus.5562

**Published:** 2019-09-04

**Authors:** Rohan Mangal, Tej G Stead, Latha Ganti, Javier Rosario

**Affiliations:** 1 Emergency Medicine, Johns Hopkins University, Baltimore, USA; 2 Emergency Medicine, Brown University, Providence, USA; 3 Emergency Medicine, Envision Physician Services, Orlando, USA; 4 Emergency Medicine, University of Central Florida College of Medicine, Orlando, USA

**Keywords:** pregnancy, appendicitis, laparoscopic appendectomy

## Abstract

The authors present a case of acute appendicitis during a first trimester pregnancy. Appendicitis in pregnancy is especially dangerous because perforation of the appendix increases the likelihood of maternal and fetal morbidity significantly. For this reason, it is important to diagnose and treat suspected appendicitis in pregnancy as soon as possible. The patient was diagnosed with appendicitis via a transabdominal ultrasound. She was provided antibiotics and underwent a laparoscopic appendectomy and recovered without complications.

## Introduction

Appendicitis is the most common nonobstetric complication during pregnancy resulting in operation, occurring at a rate of one in every 500-2000 pregnancies [1-2]. However, an appendicitis during pregnancy is also more susceptible to complications. Most notably, pregnant patients are at greater risk of appendix perforation, 43% in pregnant women versus 4%-19% in general population [3]. Perforation presents serious risk for intraperitoneal infections and has shown a greater incidence for maternal and fetal death [4].

## Case presentation

A 21-year-old female, who was eight weeks pregnant with her first child, presented to the ED complaining of right lower quadrant pain. Her pain began rather abruptly while she was at work, and was associated with nausea. Her vital signs in the ED were temperature 37°C, pulse 110 beats per minute, respirations 18 per minute, and blood pressure 122/81 mmHg, with oxygen saturation of 99% on room air. 

Physical examination confirmed tenderness in the lower right quadrant with guarding and rebound. Remainder of abdominal exam was negative. Laboratory evaluation demonstrated leukocytosis with a white blood cell count of 19.89 x 10^9^ cells per liter (normal range 4-11 x 10^9 ^cells/L), with 81% neutrophils (normal range 37%-74%), indicating neutrophilia. Her chemistries were normal. 

**Figure 1:**
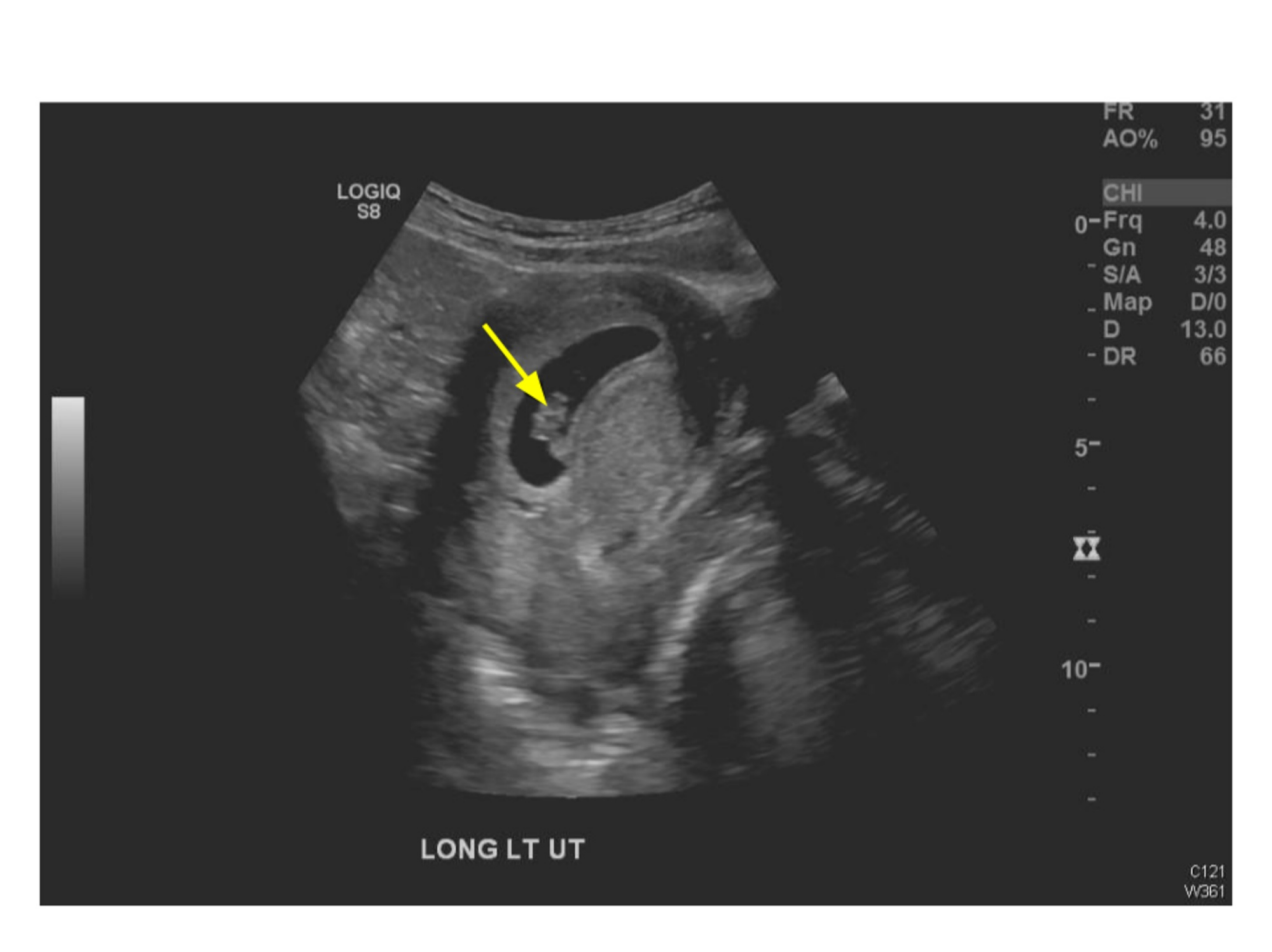
Sonogram demonstrating an intrauterine pregnancy.

**Figure 2:**
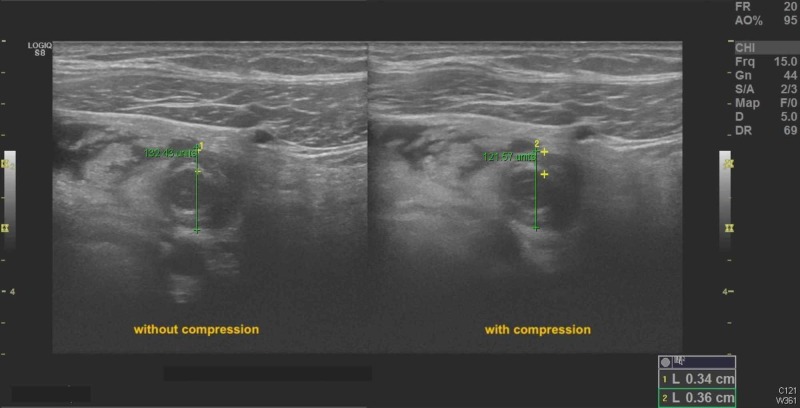
Sonogram demonstrating minimal compressibility of appendix on transverse view. Note the depth of the noncompressed and compressed appendix are almost equal.

**Figure 3:**
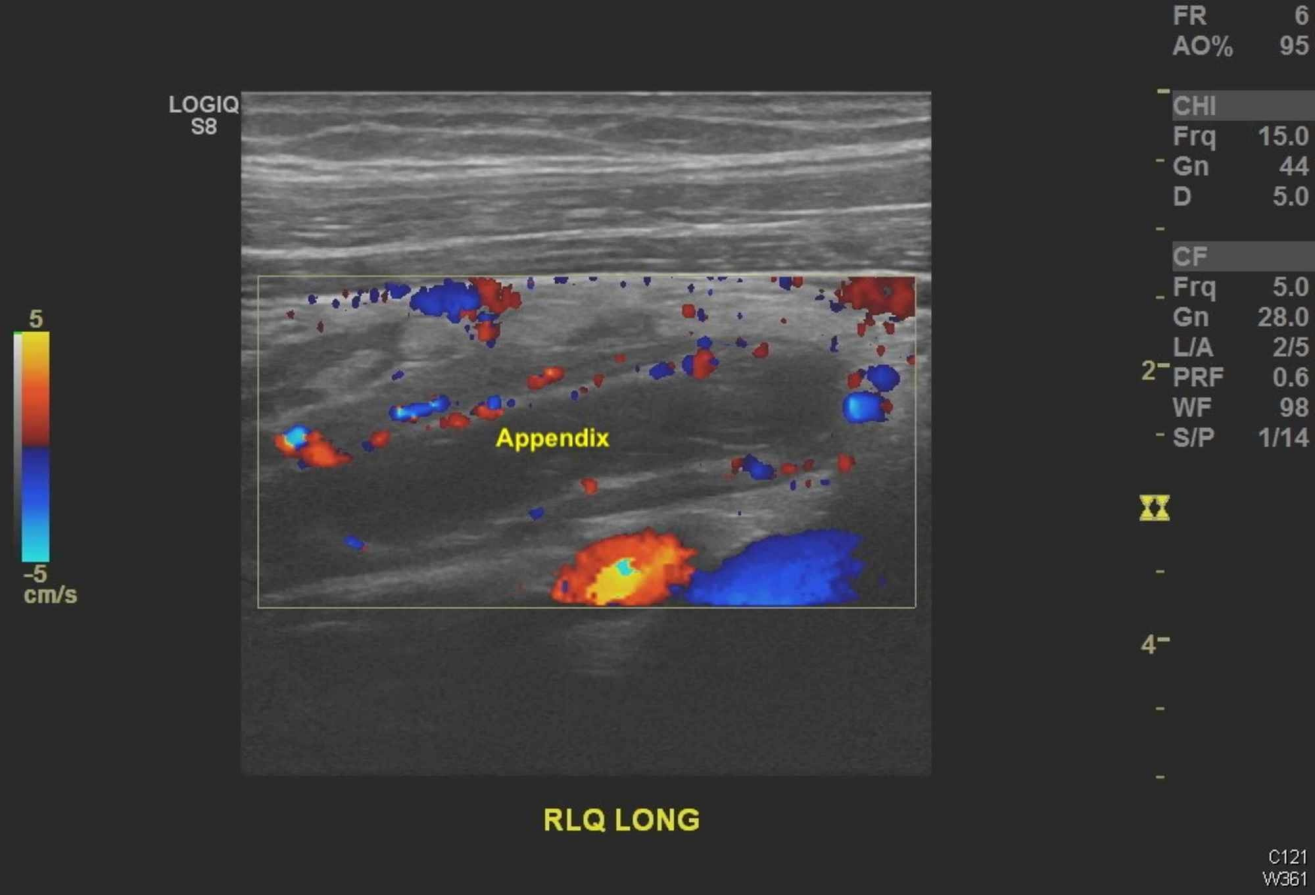
Sonogram demonstrating a long axis view of the appendix, a tubular structure that appears elongated beyond normal.

Transabdominal ultrasound (US) revealed an intrauterine gestational sac containing a fetal pole, evaluating the gestation at eight weeks, one day (Figure 1). There was blood flow to both ovaries and no adnexal lesions visible. A noncompressible fluid-filled, slightly hypervascular tubular structure was located in the right lower quadrant of the abdomen, measuring 16 mm in diameter with rebound tenderness, confirming acute appendicitis (Figures 2-3).

She received 3.375 g intravenous piperacillin-tazobactam and was taken to the operating room for a laparoscopic appendectomy. She did well postoperatively and had no complications. She was seen by the obstetric service the next day, and the pregnancy remained intact. Two weeks following her surgery, repeat ultrasonography demonstrated a properly progressing pregnancy with good fetal heart tones.

## Discussion

A timely diagnosis is important for lowering risk of appendix perforation. Appendicitis diagnosis in pregnancy can become more convoluted the later the pregnancy stage. In our patient, the symptoms were relatively classic, and her slender body habitus made sonographic evaluation straight forward. A review of 22 cases shows that typical symptoms of appendicitis, including nausea, vomiting, and fever, are unreliable in pregnant patients with appendicitis who report abdominal pain [5]. Thus, an ultrasound may be necessary to confirm an appendicitis in expecting mothers. 

Following diagnosis and the decision to operate, physicians decide between an open or laparoscopic surgery. In a review of 45 cases, 15 (33%) had a successful laparoscopic appendectomy in the first trimester, while 22 (49%) did in the second trimester-thus this operative technique is most often recommended within 26 weeks of pregnancy [6]. A meta-analysis [7] covering 905 laparoscopic appendectomies and 3789 open appendectomies found no significant difference in the rates of fetal loss or preterm delivery between open and laparoscopic appendectomy.

Ultrasonography is a highly useful imaging modality for diagnosing acute appendicitis. In a review of 181 patients with acute appendicitis, appendix diameter was found to be highly predictive. Appendicitis was present in 2.6% of cases when diameter was ≤ 6 mm, 65% of cases when between 6 and 8 mm, and 96% of cases when ≥ 8 mm [8]. Other signs include noncompressibility of the appendix and wall thickness ≥ 3mm. Overall, in a review of 9121 patients across 25 studies, US had a sensitivity of 83.7%, specificity of 95.9%, and an accuracy of 92.2% in diagnosing acute appendicitis [9]. Sometimes US is inconclusive for appendicitis due to patient body habitus or intestinal gas. In these situations, MRI is the imaging modality of choice, with sensitivities and specificities of 80%-100% and 93%-98%, respectively [10].

In our patient’s case, the appendix diameter was almost double the upper-end of diagnostic values, and the patient was taken expeditiously to the operating room without any further studies. This was extremely important to prevent rupture of the appendix which would have resulted in increased morbidity to both mother and the fetus. Up to 26% of first trimester pregnancies result in miscarriage [11], and 43% of women report having one or more first trimester miscarriages [12]. Given this baseline risk, our patient was at increased risk of miscarriage due to the appendicitis and ensuing surgery. However, due to the prompt action by the clinical teams, the patient and fetus did well. Somewhat unique to this case is the ability for us to follow the patient, and being able to document a healthy pregnancy both immediately postop as well as two weeks afterward.

## Conclusions

Appendicitis is not an uncommon complication during pregnancy. A timely ultrasound can confirm the diagnosis of appendicitis. Measures should be taken to avoid appendix perforation, as this can lead to fetal mortality. A laparoscopic appendectomy should be considered for treating related cases of appendicitis during pregnancy.
